# Supplementary material for: Determinants of sustained stabilization of beta-cell function following short-term insulin therapy in type 2 diabetes
Source: Nat Commun. 2023 Jul 27;14:4514. doi: 10.1038/s41467-023-40287-w (PMC10374648; doi:10.1038/s41467-023-40287-w)
Supplement: Supplementary file 1 — Supplementary Information [file 41467_2023_40287_MOESM1_ESM.pdf]

Supplementary Information

Supplementary Figure 1: Daily profile of mean capillary blood glucose during induction IIT in those with and without subsequent stabilization of beta-cell function at 2-years. The number of participants in each group is indicated by n.

Supplementary Figure 2: Kaplan-Meier curves showing time to initial loss of stabilization of beta-cell function between the metformin and metformin + IIT arms (defined by 2 consecutive visits with ISSI-2 lower than at baseline)

Supplementary Figure 3: Glucose response on the OGTT in the stabilization and non-stabilization groups at (A) baseline, (B) 3-weeks, (C) 6-months, (D) 12-months, (E) 18-months, and (F) 24-months. The number of participants in each group is indicated by n.

Suppl Figure 1: Daily profile of mean capillary blood glucose during induction IIT in those with and without subsequent stabilization of beta-cell function at 2-years. The number of participants in each group is indicated by n.

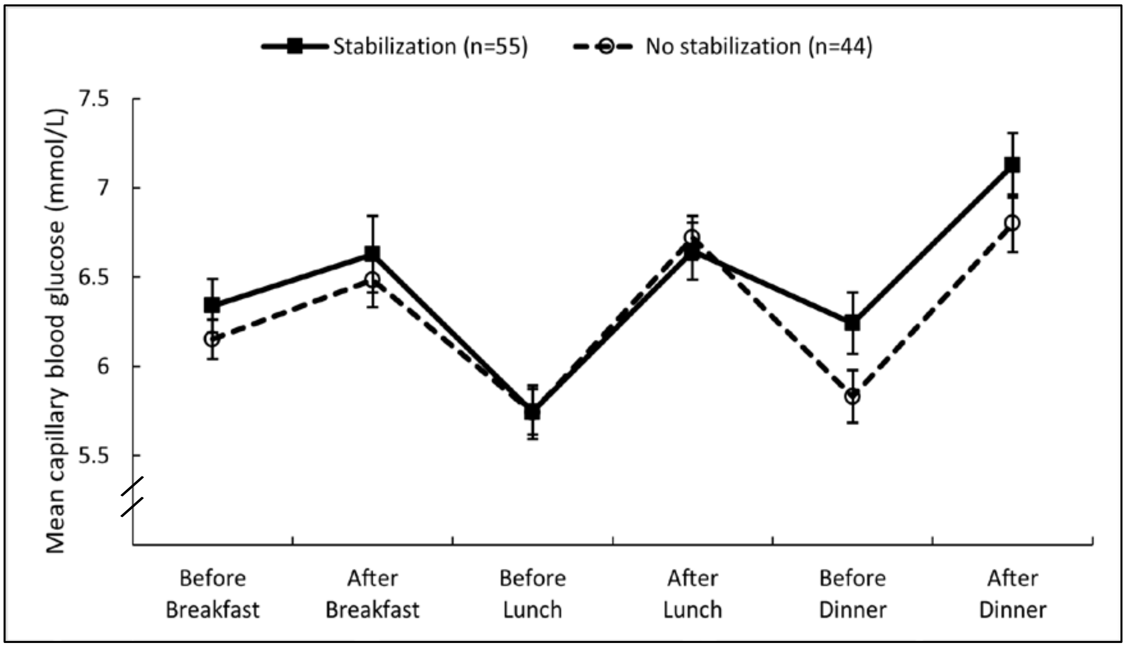

Data are presented as mean glucose ± standard error.

Glucose measurements after meals are 2-hour post-meal

Number of participants: no stabilization n=44; stabilization n=55.

Suppl Fig 2: Kaplan-Meier curves showing time to initial loss of stabilization of beta-cell function between the metformin and metformin + IIT arms (defined by 2 consecutive visits with ISSI-2 lower than at baseline)

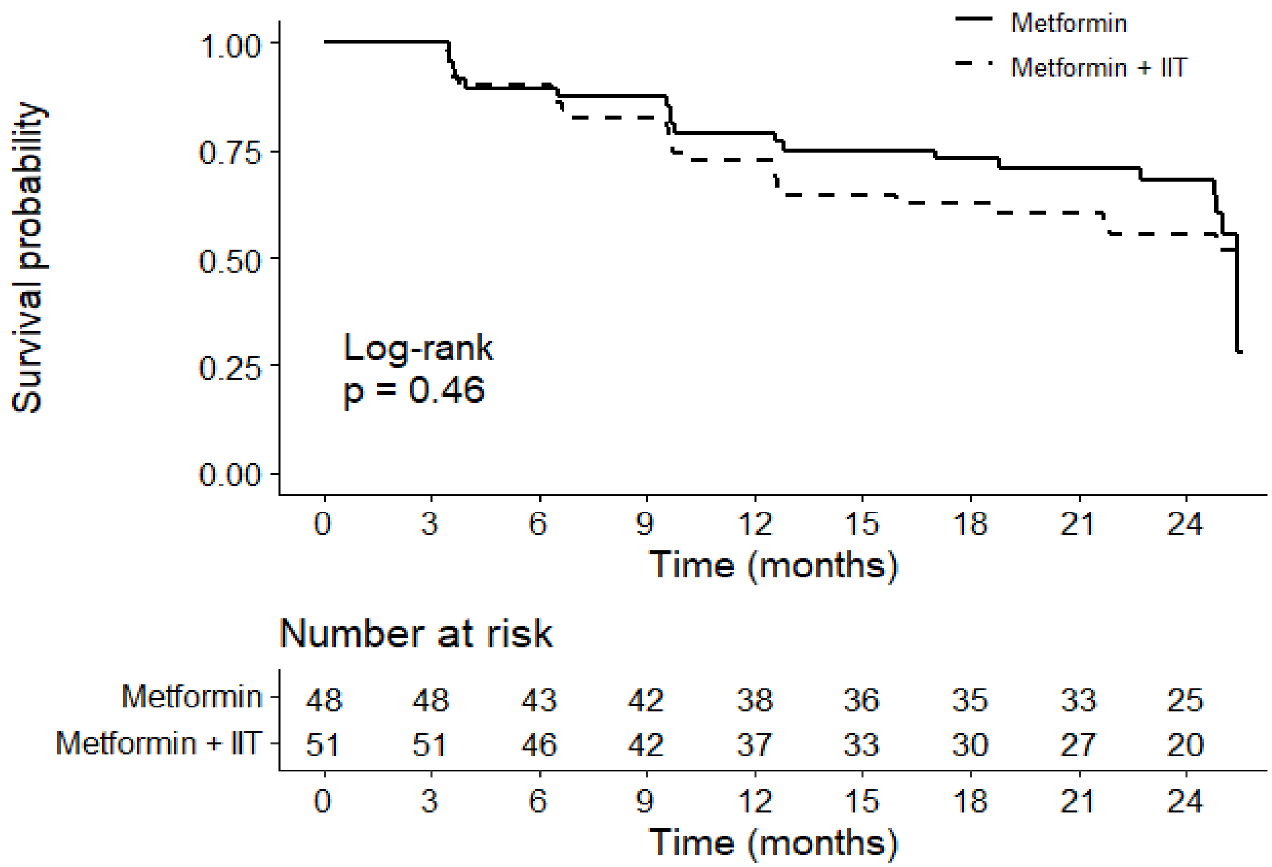

Suppl Fig. 3: Glucose response on the OGTT in the stabilization and non-stabilization groups at (A) baseline, (B) 3-weeks, (C) 6-months (D) 12-months, (E) 18-months, and (F) 24-months. The number of participants in each group is indicated by n.

Panel A: At baseline

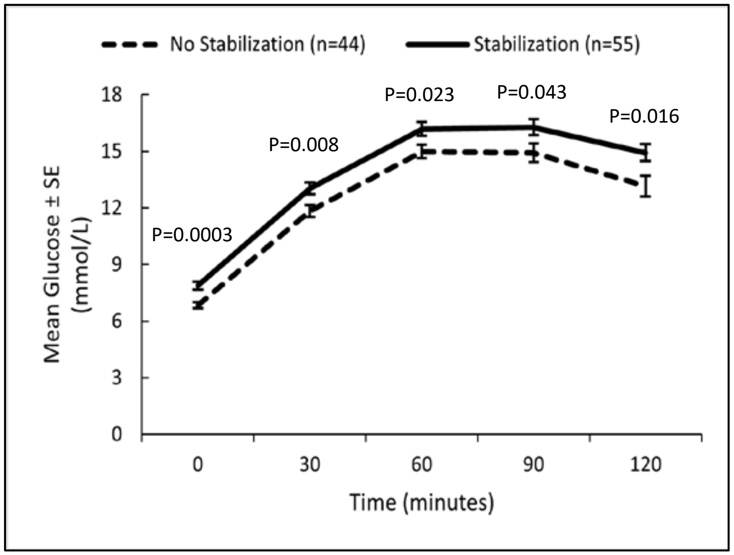

Panel B: At 3-weeks

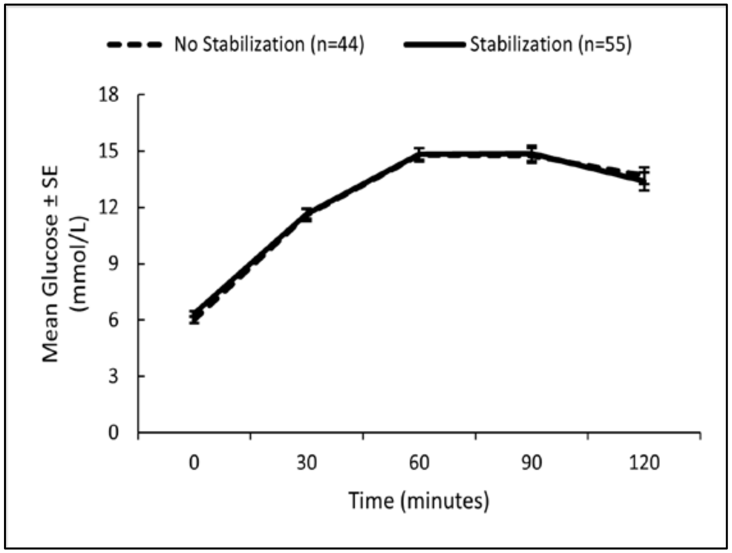

Panel C: At 6-months

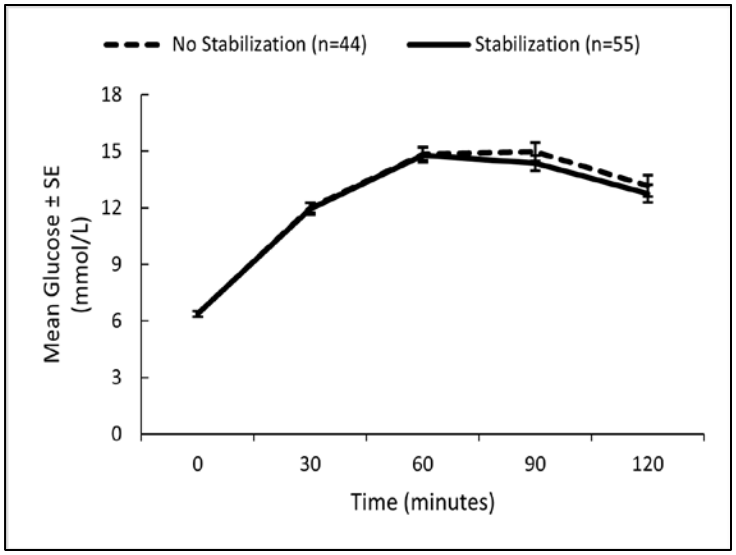

Panel D: At 12-months

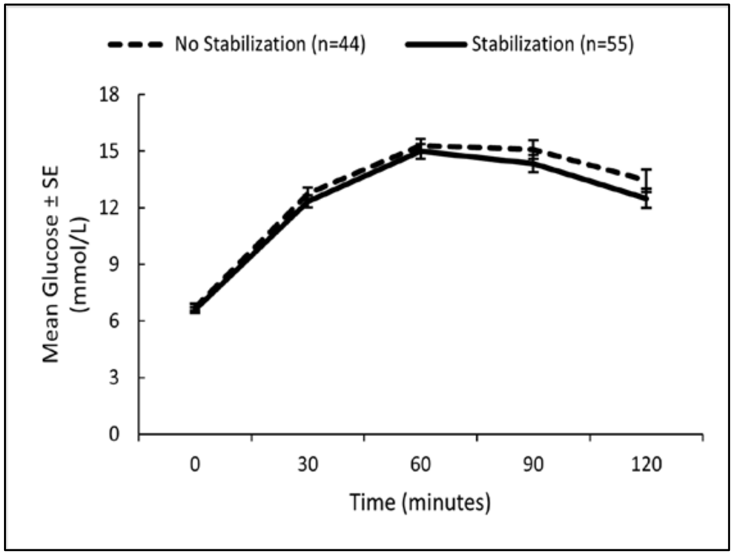

Panel E: At 18-months

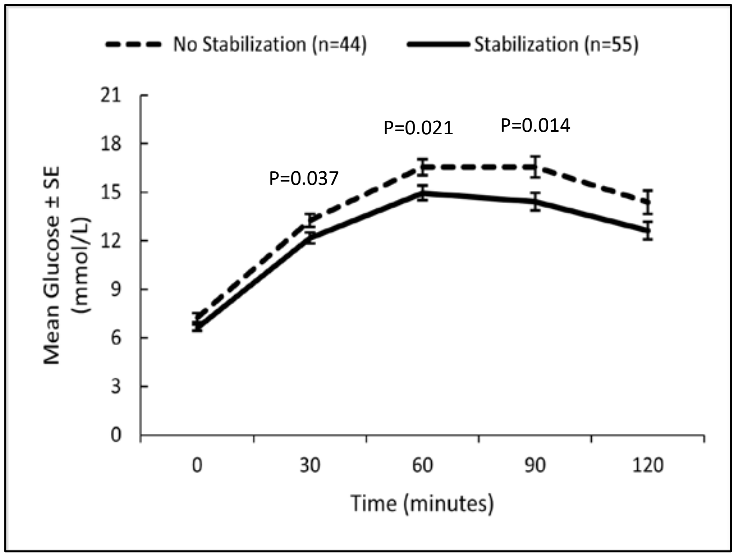

Panel F: At 24-months

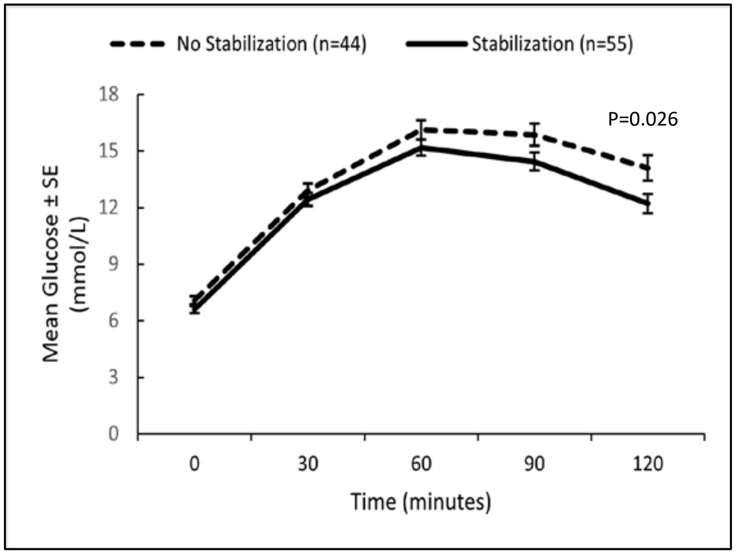

Data presented as mean ± standard error. Two-sided t-tests were performed for the mean difference between the groups, with no adjustment for multiple comparisons. Number of participants: no stabilization n=44; stabilization n=55.
