# Supplementary material for: Determinants of sustained stabilization of beta-cell function following short-term insulin therapy in type 2 diabetes
Source: Nat Commun. 2023 Jul 27;14:4514. doi: 10.1038/s41467-023-40287-w (PMC10374648; doi:10.1038/s41467-023-40287-w)
Supplement: Supplementary file 3 — Reporting Summary [file 41467_2023_40287_MOESM3_ESM.pdf]

nature portfolio

Corresponding author(s): Prof Ravi Retnakaran

Last updated by author(s): Jul 15, 2023

Reporting Summary

Nature Portfolio wishes to improve the reproducibility of the work that we publish. This form provides structure for consistency and transparency in reporting. For further information on Nature Portfolio policies, see our Editorial Policies and the Editorial Policy Checklist.

Statistics

For all statistical analyses, confirm that the following items are present in the figure legend, table legend, main text, or Methods section.

n/a	Confirmed	
☐	☒	The exact sample size (n) for each experimental group/condition, given as a discrete number and unit of measurement
☒	☐	A statement on whether measurements were taken from distinct samples or whether the same sample was measured repeatedly
☐	☒	The statistical test(s) used AND whether they are one- or two-sided<br>*Only common tests should be described solely by name; describe more complex techniques in the Methods section.*
☐	☒	A description of all covariates tested
☐	☒	A description of any assumptions or corrections, such as tests of normality and adjustment for multiple comparisons
☐	☒	A full description of the statistical parameters including central tendency (e.g. means) or other basic estimates (e.g. regression coefficient) AND variation (e.g. standard deviation) or associated estimates of uncertainty (e.g. confidence intervals)
☐	☒	For null hypothesis testing, the test statistic (e.g. F, t, r) with confidence intervals, effect sizes, degrees of freedom and P value noted<br>*Give P values as exact values whenever suitable.*
☒	☐	For Bayesian analysis, information on the choice of priors and Markov chain Monte Carlo settings
☒	☐	For hierarchical and complex designs, identification of the appropriate level for tests and full reporting of outcomes
☐	☒	Estimates of effect sizes (e.g. Cohen's d, Pearson's r), indicating how they were calculated

Our web collection on statistics for biologists contains articles on many of the points above.

Software and code

Policy information about availability of computer code

Data collection	Data from study visits were maintained in study charts and entered into an Access database. De-identified data from the resultant dataset can be made available under restricted access from the corresponding author, for academic purposes, subject to a material transfer agreement and approval of the Mount Sinai Hospital Research Ethics Board.
Data analysis	Statistical analyses were conducted with R 4.2.2.

For manuscripts utilizing custom algorithms or software that are central to the research but not yet described in published literature, software must be made available to editors and reviewers. We strongly encourage code deposition in a community repository (e.g. GitHub). See the Nature Portfolio guidelines for submitting code & software for further information.

Data

Policy information about availability of data

All manuscripts must include a data availability statement. This statement should provide the following information, where applicable:

- Accession codes, unique identifiers, or web links for publicly available datasets
- A description of any restrictions on data availability
- For clinical datasets or third party data, please ensure that the statement adheres to our policy

De-identified data can be made available under restricted access from the corresponding author (Ravi.Retnakaran@SinaiHealth.ca), for academic purposes, subject to a material transfer agreement and approval of the Mount Sinai Hospital Research Ethics Board. Individual participant data that underlie the results reported in this article can be made available by this mechanism, after de-identification, to achieve the aims in the approved proposal. Access is controlled in this way because

of the clinical nature of the data. The study protocol can also be made available in this way. This data access mechanism will be available beginning 9 months and ending 36 months following publication of this article. We will attempt to respond to requests within 3 months, pending Research Ethics Board capacity to do so within this time frame. Source data for figures have been provided with this paper.

Research involving human participants, their data, or biological material

Policy information about studies with human participants or human data. See also policy information about sex, gender (identity/presentation), and sexual orientation and race, ethnicity and racism.

Reporting on sex and gender	Self-reported data on sex were collected and post-hoc exploratory analyses were performed to determine if sex influenced the findings. It should be noted, however, that these were post-hoc analyses, recognizing that the sample size was not designed to be large enough for robust sex-based analyses. Data were not collected on gender, as a potential effect of gender role on the impact of the interventions was not hypothesized a priori.
Reporting on race, ethnicity, or other socially relevant groupings	Self-reported data on ethnicity were collected and post-hoc exploratory analyses were performed to determine if ethnicity influenced the findings. It should be noted, however, that these were post-hoc analyses, recognizing that the sample size was not designed to be large enough for robust ethnicity-based analyses.
Population characteristics	The trial population consisted of 108 adults with T2DM of median 1.3-years duration (interquartile range 0.5–3.0 years), mean age 59.2 ±17.9 years, and mean baseline A1c 6.6 ±0.6%.
Recruitment	Participants were recruited from the practices of family physicians (either by screening charts or physician referral) and in response to advertising of the study. While self-selection can always potentially introduce a bias in clinical trial participation (i.e. since not all people will want to participate in a 2 year trial), it is unlikely to have affected the determinants of sustained stabilization of beta-cell function as reported in this manuscript (i.e. reversible beta-cell dysfunction, hepatic insulin sensitivity).
Ethics oversight	This multi-centre clinical trial was approved by the research ethics boards of Mount Sinai Hospital (Toronto, ON), Western University (London, ON), and Hamilton Health Sciences (Hamilton, ON).

Note that full information on the approval of the study protocol must also be provided in the manuscript.

Field-specific reporting

Please select the one below that is the best fit for your research. If you are not sure, read the appropriate sections before making your selection.

☒ Life sciences ☐ Behavioural & social sciences ☐ Ecological, evolutionary & environmental sciences

For a reference copy of the document with all sections, see nature.com/documents/nr-reporting-summary-flat.pdf

Life sciences study design

All studies must disclose on these points even when the disclosure is negative.

Sample size	The pre-trial power calculation indicated that a sample size of 88 participants (44 in each arm) would provide 80% power to detect a 20% difference in baseline-adjusted ISSI-2 at 2-years between the MET and MET+IIT arms (based on estimated mean baseline-adjusted ISSI-2 at 2-years of 233 and 280, respectively) at significance level (alpha) of 0.05, with standard deviation 100 and assuming a correlation of 0.6 between ISSI-2 at baseline and study end.
Data exclusions	Of the 108 study participants, there were 9 individuals whose last study visit occurred at <12-months, representing a duration of follow-up that was considered insufficient for determination of sustained stabilization of beta-cell function. The current analysis was thus performed in the 99 participants in whom sustained stabilization of beta-cell function could be assessed.
Replication	A series of sensitivity analyses were performed to confirm robustness of the findings, including analyses in all participants who completed at least 1 study visit after receiving any maintenance therapy (i.e. completed at least the 3-month visit) (n=105). These analyses confirmed the findings reported in the manuscript.
Randomization	At baseline, participants were randomized to the treatment arms in a 1:1 manner. The Applied Health Research Centre (Toronto) prepared the computer-generated random allocation sequence in variable permuted blocks of sizes 2 and 4, and provided the allocation to the investigators in sealed envelopes that were opened at randomization.
Blinding	This trial had an open-label design since masking of periodic intensive insulin therapy (IIT) would be impractical. As some protection against bias, the primary outcome of beta-cell function is a physiologic measure that is not readily amenable to manipulation by either participants or providers, and all biochemical analyses were performed by personnel who were unaware of treatment allocation.

Reporting for specific materials, systems and methods

We require information from authors about some types of materials, experimental systems and methods used in many studies. Here, indicate whether each material, system or method listed is relevant to your study. If you are not sure if a list item applies to your research, read the appropriate section before selecting a response.

Materials & experimental systems

n/a	Involved in the study
☒	☐ Antibodies
☒	☐ Eukaryotic cell lines
☒	☐ Palaeontology and archaeology
☒	☐ Animals and other organisms
☐	☒ Clinical data
☒	☐ Dual use research of concern
☒	☐ Plants

Methods

n/a	Involved in the study
☒	☐ ChIP-seq
☒	☐ Flow cytometry
☒	☐ MRI-based neuroimaging

Clinical data

Policy information about clinical studies

All manuscripts should comply with the ICMJEguidelines for publication of clinical research and a completedCONSORT checklist must be included with all submissions.

Clinical trial registration	ClinicalTrials.Gov NCT02192424
Study protocol	The study protocol has been enclosed with submission of the manuscript. The mechanism for access to the protocol is described in the Data Availability Statement.
Data collection	The study population was recruited between 28/07/2014 and 20/06/2018. The study was completed in Sep 2020. This multi-centre trial took place at Mount Sinai Hospital (Toronto, Ontario, Canada), Western University (London, Ontario, Canada), and Hamilton Health Sciences (Hamilton, Ontario, Canada), as noted in the first line of the Methods section of the manuscript.
Outcomes	The pre-specified primary and secondary outcomes of the trial were previously reported in reference 11, as described in the Introduction and in the first paragraph of the Results section of the current manuscript. The current manuscript reports secondary analyses of the trial. The main outcome of the current analysis was sustained stabilization of beta-cell function, which was defined by having higher ISSI-2 at 2-years (or last study visit) than at baseline. This information is provided in the Outcomes section of the Methods.
